# Preoperative gamma-glutamyl transferase to lymphocyte ratio predicts recurrence in non-muscle-invasive bladder cancer

**DOI:** 10.3389/fonc.2026.1724968

**Published:** 2026-02-10

**Authors:** Xueqiao Zhang, Shiqiang Su, Lizhe Liu, Feifan Song, Xiongjie Cui, Yunpeng Cao, Chao Li, Shen Li, Hanxing He, Yuanhui Kang, Jin Zhang

**Affiliations:** 1 Department of Urology, The People’s Hospital of Shijiazhuang, Shijiazhuang, Hebei, China; 2 Graduate School, Hebei Medical University, Shijiazhuang, Hebei, China; 3 Institute of Medicine and Health, Hebei Medical University, Shijiazhuang, China

**Keywords:** gamma-glutamyl transferase to lymphocyte ratio, non-muscle-invasive bladder cancer, predictive indicator, prognosis, tumor recurrence

## Abstract

**Background:**

The prognostic value of the preoperative gamma-glutamyl transferase to lymphocyte ratio (GLR), an established marker in many solid tumors, remains unclear in non-muscle-invasive bladder cancer (NMIBC). This study aimed to investigate the significance of GLR for predicting recurrence in NMIBC patients after transurethral resection of bladder tumor (TURBt).

**Methods:**

We retrospectively analyzed 254 patients with primary NMIBC who underwent TURBt from 2013 to 2024. Preoperative GLR was calculated from blood tests performed within one week of surgery. The primary endpoint was recurrence-free survival (RFS). The optimal GLR cutoff was determined using receiver operating characteristic (ROC) curve analysis. Kaplan-Meier method, log-rank tests, and Cox proportional hazards models were used to assess survival outcomes and identify independent prognostic factors. A novel prognostic nomogram for RFS was constructed and its performance was evaluated by concordance index (C-index), calibration curves, time-dependent ROC, and decision curve analysis (DCA).

**Results:**

The optimal GLR cutoff was identified as 11.71. Patients with high GLR (> 11.71) had significantly poorer RFS (P < 0.001). On multivariate analysis, a high GLR was an independent predictor of postoperative recurrence (Hazard Ratio (HR) = 2.822, 95% Confidence Interval (CI): 1.651–4.824, P < 0.001). A nomogram incorporating GLR and established clinicopathological factors was developed. The inclusion of GLR significantly improved the model’s predictive accuracy, increasing the C-index from 0.745 to 0.785. The nomogram demonstrated good calibration and discrimination (3-year Area Under the Curve (AUC) = 0.72) and provided superior net clinical benefit in DCA. The prognostic value of GLR remained robust across all clinicopathological subgroups.

**Conclusion:**

Preoperative GLR is a simple, cost-effective, and reliable independent biomarker for predicting recurrence in NMIBC patients following TURBt. The GLR-based nomogram integrates systemic inflammation with clinical risk factors, offering a more precise tool for individualized risk stratification. This model can help guide personalized follow-up strategies and adjuvant treatment decisions, holding significant potential for clinical application.

## 1 Introduction

Bladder cancer (BCa) is a globally prevalent malignancy, ranking as the ninth most common cancer by incidence and the second leading malignancy within the urological system (1). Projections for the United States alone estimate approximately 84,870 new cases and 33,140 deaths from BCa in 2025 (2). Clinically, about 75% of cases are initially diagnosed as non-muscle-invasive bladder cancer (NMIBC). Although patients with NMIBC have a relatively favorable prognosis, they face a significant clinical challenge due to high recurrence rates and a notable risk of progression (3). The standard-of-care for NMIBC is transurethral resection of bladder tumor (TURBt), often followed by intravesical instillation therapy to mitigate recurrence. Nevertheless, despite standard treatment, up to 50–70% of patients experience tumor recurrence within five years, and 10–30% progress to the more aggressive muscle-invasive bladder cancer (MIBC) (4).

Currently employed prognostic models, such as the scoring systems from the European Organisation for Research and Treatment of Cancer (EORTC) and the Spanish Urological Club for Oncological Treatment (CUETO), primarily rely on clinicopathological features—including tumor size, number, grade, and stage—to stratify the risk of recurrence and progression (3, 5). While these models provide valuable guidance, their predictive accuracy remains suboptimal, particularly for individualized risk assessment. Emerging strategies based on molecular subtyping, such as the BCG response subtype (BRS) stratification method and models involving N6-methyladenosine (m6A)-related long non-coding RNAs, have demonstrated superior predictive efficacy. However, their routine clinical implementation is hampered by technical complexity and high costs (6, 7). Consequently, the identification of an accessible, cost-effective, and reproducible biomarker to improve preoperative risk stratification in NMIBC remains a critical unmet need.

Inflammation and metabolic reprogramming are recognized as two core hallmarks of cancer (8). Gamma-glutamyl transferase (GGT) is a cell membrane-bound enzyme that plays a critical role in maintaining cellular redox homeostasis by catalyzing the degradation of glutathione (GSH). Within the tumor microenvironment, elevated oxidative stress often leads to the upregulation of GGT expression. This not only supplies tumor cells with precursors for GSH synthesis, thereby enhancing their antioxidant capacity and therapeutic resistance, but also reflects systemic metabolic dysregulation (9). A substantial body of evidence has established that elevated serum GGT levels are an adverse prognostic marker in various malignancies, including breast, hepatocellular, and prostate cancers (10–13). Concurrently, lymphocytes are the central effector cells of the host’s anti-tumor immunity, and their count and functional status serve as a direct indicator of immune surveillance capabilities. Lymphopenia is frequently associated with compromised immune function, which allows tumor cells to evade immune clearance and thereby promotes tumor recurrence and progression (14).

Building on this rationale, a composite index that integrates metabolic stress (GGT) and immune status (lymphocytes)—the GLR—may more comprehensively reflect the complex biological interplay of host-tumor interactions than either marker alone. In recent years, GLR has emerged as a novel prognostic biomarker, demonstrating superior predictive value in various cancers, such as non-functional pancreatic neuroendocrine tumors, hepatocellular carcinoma, and oral cancer (15–17). However, its prognostic significance in patients with NMIBC has yet to be elucidated.

Therefore, this study aimed to be the first to evaluate the role of preoperative GLR in predicting postoperative recurrence in NMIBC patients undergoing TURBt. Furthermore, we sought to investigate whether incorporating GLR into a prognostic nomogram could enhance its predictive performance, with the ultimate goal of providing a new, evidence-based tool for the individualized management of NMIBC.

## 2 Materials and methods

### 2.1 Study population

**Figure 1:**
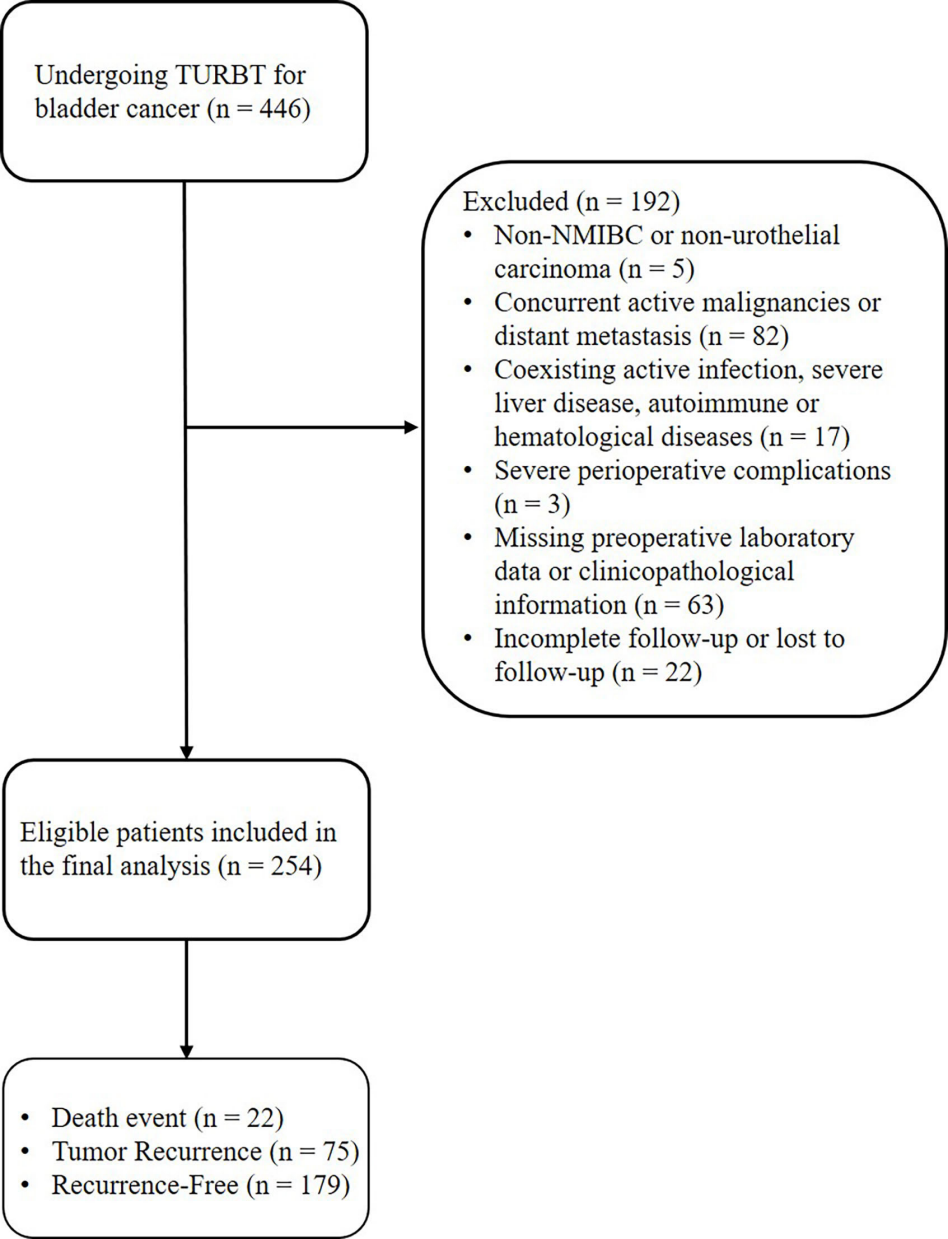
The flow chart for patient selection. TURBT, transurethral resection of bladder tumor; NMIBC, non-muscle-invasive bladder cancer.

This study was a retrospective cohort study. We consecutively enrolled patients who underwent TURBt and were pathologically diagnosed with primary NMIBC for the first time at Shijiazhuang People’s Hospital between November 2013 and January 2024. The inclusion criteria were as follows: (1) pathologically confirmed primary NMIBC; (2) underwent complete TURBt surgery; and (3) had complete clinical, pathological, and follow-up data. The exclusion criteria were: (1) presence of distant metastasis or secondary bladder tumors; (2) active preoperative infection or severe liver disease (e.g., viral hepatitis, cirrhosis); (3) coexisting autoimmune or hematological diseases; (4) histology other than urothelial carcinoma of the bladder (UCB); (5) missing key preoperative laboratory data (GGT, lymphocyte count) or clinicopathological information; (6) incomplete follow-up data or lost to follow-up; (7) concurrent active malignancies; and (8) severe perioperative complications. Based on these criteria, a total of 254 patients were ultimately included in this study. Among the 254 eligible patients, 75 (29.5%) experienced tumor recurrence, and 179 (70.5%) remained recurrence-free during the follow-up period. A total of 22 patients died (**Figure 1**). The study protocol was approved by the Ethics Committee of Shijiazhuang People’s Hospital and was conducted in accordance with the principles of the Declaration of Helsinki.

Clinical baseline data, including age, sex, smoking history, and comorbidities, were collected from the electronic medical record system. All pathological slides were independently reviewed by two senior pathologists. Tumor grade was determined according to the 2004/2016 World Health Organization (WHO) classification (18), and tumor stage was assigned using the 8th edition of the American Joint Committee on Cancer (AJCC) TNM staging system (2017) (19).

### 2.2 Calculation of GLR, NLR, and PLR

Venous blood samples were collected from all patients within one week prior to surgery after fasting for 8–12 hours. Serum GGT concentration (U/L), Absolute neutrophil count (ANC), Peripheral platelet count and peripheral blood absolute lymphocyte count (×10^9/L) were measured using an automated analyzer at the hospital’s central laboratory. The GLR was calculated as follows: GLR = GGT (U/L)/Lymphocyte Count (×10^9/L). The NLR was calculated using the formula: NLR = Absolute neutrophil count (×10^9/L)/Lymphocyte count (×10^9/L). The PLR was calculated using the formula: PLR = Peripheral platelet count (×10^9/L)/Lymphocyte count (×10^9/L).

### 2.3 Postoperative adjuvant therapy

Postoperative intravesical chemotherapy utilized pirarubicin, with dosages ranging from 30 to 50 mg per instillation, individualized based on patient tolerance. An immediate single instillation was administered within 24 hours following TURBt. This was followed by an induction course of weekly instillations for 4 to 8 weeks, and subsequently, a maintenance phase of monthly instillations for 6 to 12 months. For patients with intermediate- to high-risk NMIBC and those with carcinoma *in situ* (CIS), intravesical immunotherapy with Bacillus Calmette-Guérin (BCG) was administered. The dosage was 120 mg per instillation. The BCG regimen commenced two weeks after surgery, beginning with an induction course of six weekly instillations, followed by a maintenance phase of monthly instillations for up to 3 years.

### 2.4 Follow-up and outcomes

All patients were followed up according to a standard protocol: every 3 months for the first 2 years, every 6 months from year 3 to 5, and annually thereafter. The final follow-up date was March 1, 2025. Follow-up visits included urinalysis, urine cytology, urinary system ultrasound, and cystoscopy. The primary study endpoint was recurrence-free survival (RFS), defined as the time from the date of TURBt to the first evidence of intravesical tumor recurrence or distant metastasis confirmed by imaging or cystoscopy, or to the last follow-up. The secondary endpoint was overall survival (OS), defined as the time from the date of surgery to death from any cause or the last follow-up.

### 2.5 Statistical analysis

All data analyses were performed using R software (Version 4.4.3) and Empower Stats (Version 4.2). A receiver operating characteristic (ROC) curve analysis was used to determine the optimal GLR cutoff value for predicting RFS and to evaluate its predictive performance. The area under the curve (AUC) values were calculated to compare the prognostic accuracy of GLR with established biomarkers (NLR and PLR). The cutoff was established based on the maximum Youden index (sensitivity + specificity − 1).

Continuous variables are presented as median (interquartile range, IQR) and were compared using the Mann-Whitney U test. Categorical variables are presented as frequency (percentage) and were compared using the Chi-square test or Fisher’s exact test, as appropriate.

Kaplan-Meier curves were plotted to visualize survival, and differences were compared using the log-rank test. Univariate and multivariate Cox proportional hazards regression models were used to analyze prognostic factors associated with RFS and OS, calculating hazard ratios (HRs) and their 95% confidence intervals (CIs). Variables with a P-value < 0.1 in the univariate analysis were included in the multivariate model. Independent prognostic factors with a P-value < 0.05 in the multivariate analysis were used to construct a nomogram for predicting 3- and 5-year RFS. The discrimination of the nomogram was assessed using the concordance index (C-index) and time-dependent ROC curves. Its calibration and stability were evaluated using calibration curves and internal validation with 1000 bootstrap resamples. Decision curve analysis (DCA) was employed to assess the clinical net benefit and utility of the nomogram across a range of threshold probabilities. To test the generalizability of GLR’s prognostic value, subgroup analyses were performed based on key clinicopathological variables (tumor size, number, grade, and stage). The presence of significant interactions between GLR and these variables was tested by introducing interaction terms into the Cox model. All statistical tests were two-sided, and a P-value < 0.05 was considered statistically significant.

## 3 Results

### 3.1 Patient clinical features

A total of 254 NMIBC patients were included in this study. The median age was 67 years (IQR: 29–92), and 218 (85.8%) were male. Regarding tumor stage, 179 patients (70.5%) were stage Ta, and 75 patients (29.5%) were stage T1. It is important to note that while no patients in our cohort were diagnosed with primary isolated carcinoma *in situ* (pure Tis), 5 patients (2.0%) presented with concomitant carcinoma *in situ* (CIS). All five of these cases were associated with T1 tumors and were classified as T1 stage in accordance with AJCC guidelines. The variable of “Concomitant CIS” has been explicitly included in the baseline characteristics (**Table 1**) and Cox regression analysis (**Table 2**). During the follow-up period, 75 patients (29.5%) experienced tumor recurrence and 22 patients (8.7%) died. Since the cumulative probability of both recurrence-free survival and overall survival remained well above 50% throughout the entire follow-up period, the median Recurrence-Free Survival (RFS) and median Overall Survival (OS) were not reached. ROC curve analysis identified an optimal GLR cutoff value of 11.71 for predicting RFS, with a sensitivity of 76.0% and a specificity of 62.6%. Accordingly, patients were stratified into a low GLR group (GLR ≤ 11.71, n = 130) and a high GLR group (GLR > 11.71, n = 124).

**Table 1:** Baseline characteristics of patients stratified by recurrence-free survival (RFS) status.

Characteristics	No recurrence	Recurrence	P value
	(n = 179)	(n = 75)	
Age (years) (mean ± SD)	65.3 ± 12.7	67.5 ± 11.3	0.191
GLR (mean ± SD)	12.1 ± 6.3	18.5 ± 11.4	< 0.001
NLR (mean ± SD)	2.6 ± 1.8	2.8 ± 1.8	0.194
PLR (mean ± SD)	129.2 ± 51.7	130.9 ± 42.1	0.371
Gender, n (%)			0.804
Female	26 (14.5%)	10 (13.3%)	
Male	153 (85.5%)	65 (86.7%)	
History of abdominal surgery, n (%)			0.082
No	160 (89.4%)	61 (81.3%)	
Yes	19 (10.6%)	14 (18.7%)	
Hypertension, n (%)			0.868
No	103 (57.5%)	44 (58.7%)	
Yes	76 (42.5%)	31 (41.3%)	
Diabetes, n (%)			0.791
No	155 (86.6%)	64 (85.3%)	
Yes	24 (13.4%)	11 (14.7%)	
Coronary heart disease, n (%)			0.534
No	160 (89.4%)	65 (86.7%)	
Yes	19 (10.6%)	10 (13.3%)	
Smoking, n (%)			0.463
No	123 (68.7%)	55 (73.3%)	
Yes	56 (31.3%)	20 (26.7%)	
Drinking, n (%)			0.312
No	70(39.1%)	34(45.3%)	
Yes	109(60.9%)	41(54.7%)	
Tumor number, n (%)			0.007
Single	125 (69.8%)	39 (52.0%)	
Multiple	54 (30.2%)	36 (48.0%)	
Tumor size, n (%)			0.004
≤3cm	156 (87.2%)	54 (72.0%)	
>3cm	23 (12.8%)	21 (28.0%)	
Tumor grade, n (%)			0.006
Low	117 (65.4%)	35 (46.7%)	
High	62 (34.6%)	40 (53.3%)	
Tumor stage, n (%)			< 0.001
Ta	138 (77.1%)	22 (29.3%)	
T1	41 (22.9%)	53 (70.7%)	
Concomitant CIS			0.155
No	177 (99%)	72 (96%)	
Yes	2 (1.1%)	3 (4.0%)	
Postoperative adjuvant therapy, n (%)			0.356
Intravesical chemotherapy	168(93.9%)	73(97.3%)	
BCG	11(6.1%)	2(2.7%)	

**Table 2:** Univariate and multivariate Cox regression analysis for recurrence-free survival (RFS).

Characteristic	Univariate analysis		Multivariate analysis	
	Hazard ratio (95% CI)	P value	Hazard ratio (95% CI)	P value
Gender				
Female	Reference			
Male	1.046(0.537-2.035)	0.895		
Age	1.017(0.998-1.036)	0.085	0.998(0.977-1.020)	0.870
Diabetes				
No	Reference			
Yes	1.092(0.575-2.071)	0.788		
History of abdominal surgery				
No	Reference		Reference	
Yes	2.235(1.245-4.013)	0.007	2.376(1.294-4.361)	0.005
Hypertension				
No	Reference			
Yes	0.962(0.607-1.523)	0.867		
Coronary heart disease				
No	Reference			
Yes	1.036(0.532-2.020)	0.917		
Smoking				
No	Reference			
Yes	0.798(0.478-1.331)	0.387		
Drinking				
No	Reference			
Yes	0.689(0.436-1.088)	0.110		
Tumor number				
Single	Reference		Reference	
Multiple	1.998(1.263-3.128)	0.003	1.957(1.235-3.100)	0.004
Tumor size				
≤3cm	Reference		Reference	
>3cm	2.543(1.526-4.237)	<0.001	1.689(1.002-2.849)	0.049
Tumor grade				
Low	Reference		Reference	
High	1.906(1.210-3.002)	0.005	1.883(1.181-3.004)	0.008
Tumor stage				
Ta	Reference		Reference	
T1	4.497(2.735-7.396)	<0.001	3.397(2.025-5.697)	<0.001
Concomitant CIS				
No	Reference		Reference	
Yes	3.034(0.955-9.640)	0.060	0.947(0.281-3.198)	0.931
GLR group				
≤11.71	Reference		Reference	
>11.71	3.521(2.070-5.989)	<0.001	2.872(1.672-4.934)	<0.001
Postoperative adjuvant therapy				
Intravesical chemotherapy	Reference			
BCG	0.566(0.138-2.317)	0.429		

Patient clinical features are presented in **Table 1**. Compared to the non-recurrence group, patients in the recurrence group had larger tumors (P = 0.004), a higher proportion of multiple tumors (P = 0.007), a higher proportion of high-grade tumors (P = 0.006), a higher proportion of T1 stage tumors (P < 0.001), and significantly higher GLR levels (P < 0.001). An analysis of risk factors associated with overall survival (OS) is detailed in **Supplementary Table 1**.

**Figure 2:**
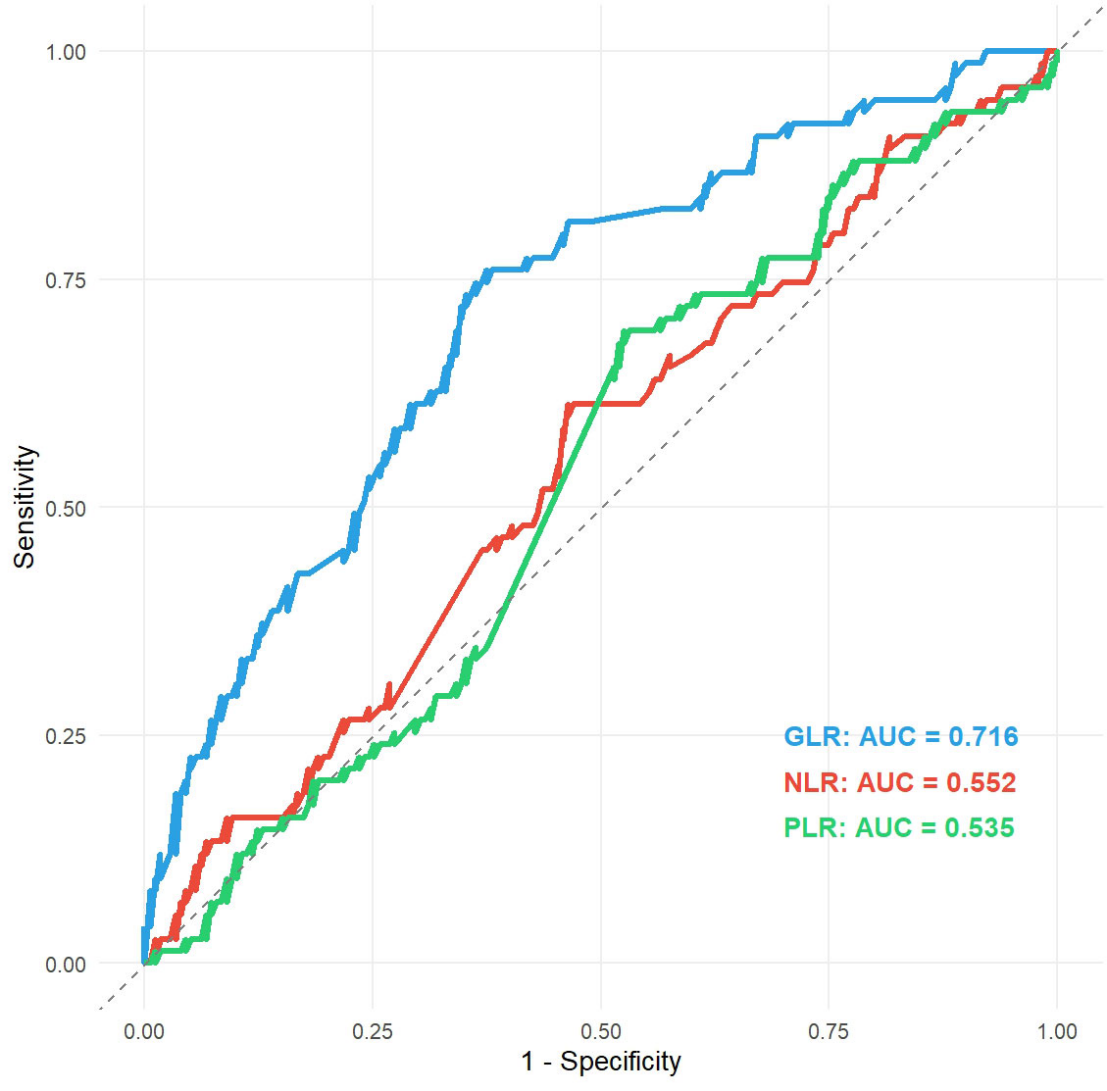
Comparison of ROC curves for GLR, NLR, and PLR. ROC, receiver operating characteristic; GLR, gamma-glutamyl transpeptidase to lymphocyte count ratio; NLR, absolute neutrophil to lymphocyte count ratio; PLR, peripheral platelet to lymphocyte count ratio.

ROC curve analysis was performed to compare the predictive value of GLR with established inflammation-based biomarkers. The GLR achieved an Area Under the Curve (AUC) of 0.716, which was notably higher than that of NLR (AUC = 0.552) and PLR (AUC = 0.535) (**Figure 2**). These results indicate that GLR possesses superior discriminative ability for predicting postoperative recurrence in NMIBC patients compared to traditional inflammatory indices.

### 3.2 Association of GLR with RFS and OS

**Figure 3:**
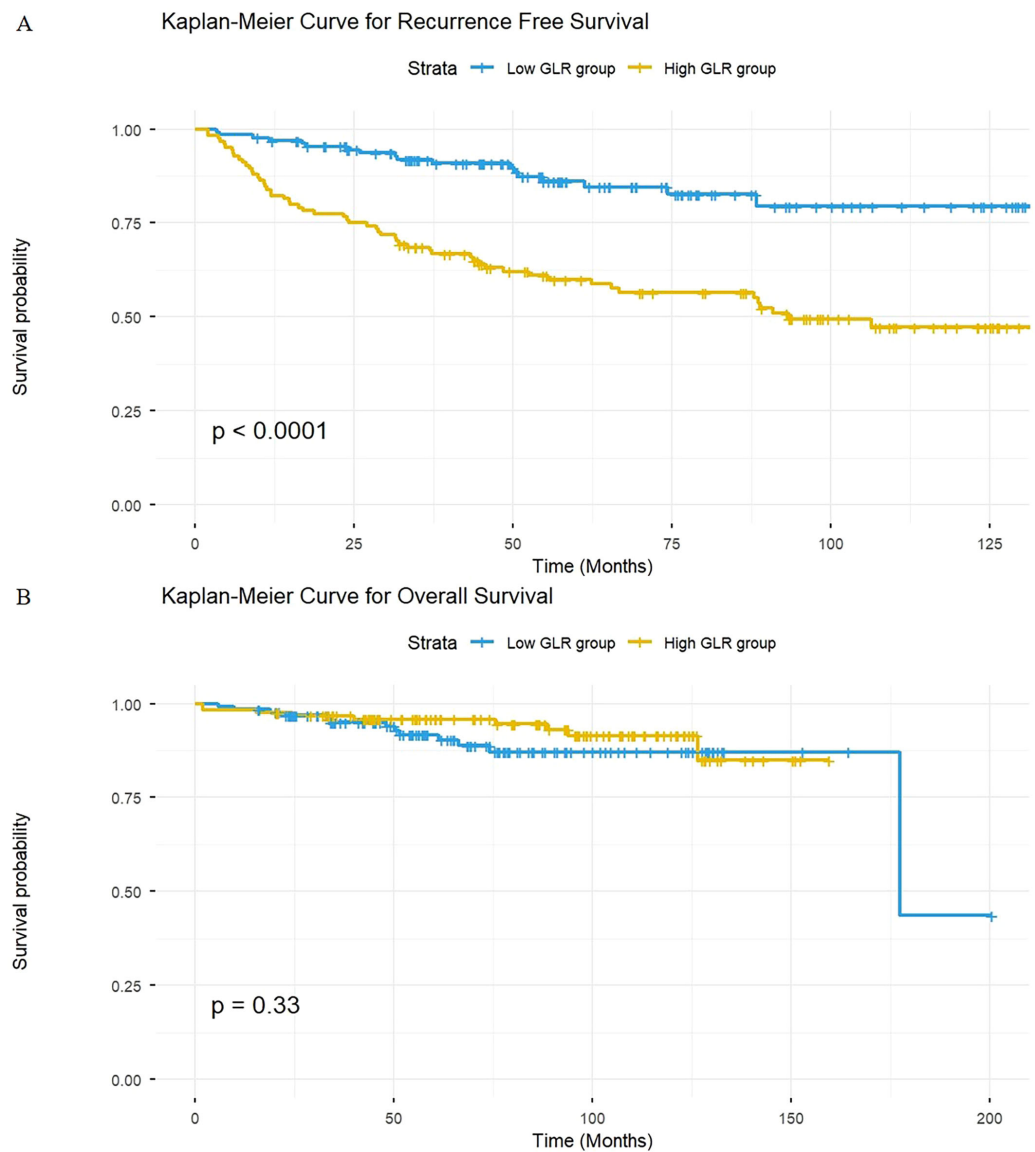
Kaplan–Meier curves for RFS **(A)** and OS **(B)** stratified by the GLR. **(A, B)** Survival curves for RFS **(A)** and OS **(B)** in all included patients. RFS, recurrence-free survival; OS, overall survival; GLR, gamma-glutamyl transpeptidase to lymphocyte count ratio.

Kaplan-Meier survival analysis revealed that patients in the high GLR group had a significantly shorter median RFS than those in the low GLR group (Log-rank P < 0.001, **Figure 3A**). However, no statistically significant difference in OS was observed between the two groups (Log-rank P = 0.33, **Figure 3B**).

In the univariate Cox regression analysis, tumor size, number, grade, stage, history of abdominal surgery, and GLR group were all significantly associated with poorer RFS (**Table 2**). After incorporating these variables, age, concomitant CIS (P < 0.1) into the multivariate Cox regression model, the results showed that a high GLR (HR = 2.872, 95% CI: 1.672–4.934, P < 0.001), history of abdominal surgery, multiple tumors, tumor size > 3 cm, high grade, and T1 stage were independent risk factors for postoperative recurrence in NMIBC patients (**Table 2**). For OS, multivariate analysis identified age and tumor grade as independent prognostic factors, whereas GLR was not significantly associated with OS (P = 0.335) (**Supplementary Table 2**).

### 3.3 Nomogram construction and performance

**Figure 4:**
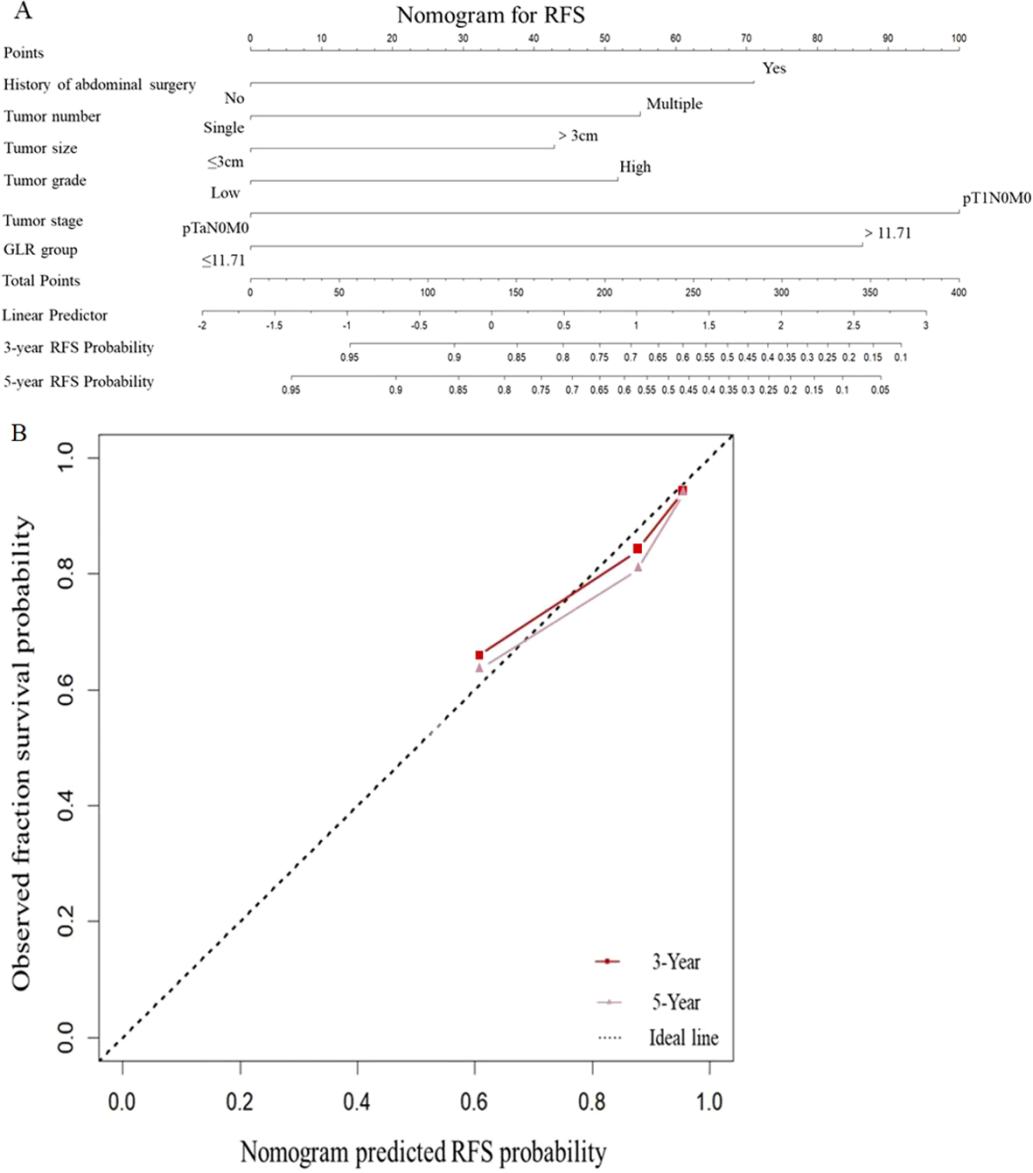
Nomograms and calibration curves for the prediction of 3- and 5-year RFS. Nomograms for 3- and 5-year RFS **(A)** prediction. Calibration curves for estimating the prediction of 3- and 5-year RFS **(B)** between the prediction and the actual observation. RFS, recurrence-free survival; GLR, gamma-glutamyl transpeptidase to lymphocyte count ratio.

Based on the six independent predictors of RFS identified from the multivariate analysis (history of abdominal surgery, tumor number, size, grade, stage, and GLR), we constructed a nomogram to predict 3- and 5-year RFS (**Figure 4A**).

The nomogram demonstrated good predictive performance. Its C-index was 0.785, which was significantly higher than that of a baseline model without GLR (C-index = 0.745), indicating that the inclusion of GLR improved the model’s discriminative ability. Internal validation using 1000 bootstrap resamples yielded a corrected C-index of 0.772, indicating good model stability (**Table 3**). The calibration curves showed good agreement between the nomogram-predicted probabilities of recurrence and the actual observed recurrence frequencies at both 3- and 5-year time points (**Figure 4B**).

**Table 3:** C-index of the nomogram for the prediction of survival outcomes.

Outcome	Models	C-index	Optimism	95% CI	Optimism-corrected C-index	ΔC-index
Recurrence-free survival	Nomogram without GLR	0.745	0.026	0.686-0.804	0.732	
	Nomogram*	0.785	0.026	0.734-0.836	0.772	
	Nomogram without GLR vs Nomogram					0.040

*History of abdominal surgery, Tumor number, Tumor size, Tumor grade, Tumor stage, GLR group were included in the nomogram for Recurrence-free survival prediction.

GLR, gamma-glutamyl transpeptidase to lymphocyte count ratio; CI, confidence interval.

**Figure 5:**
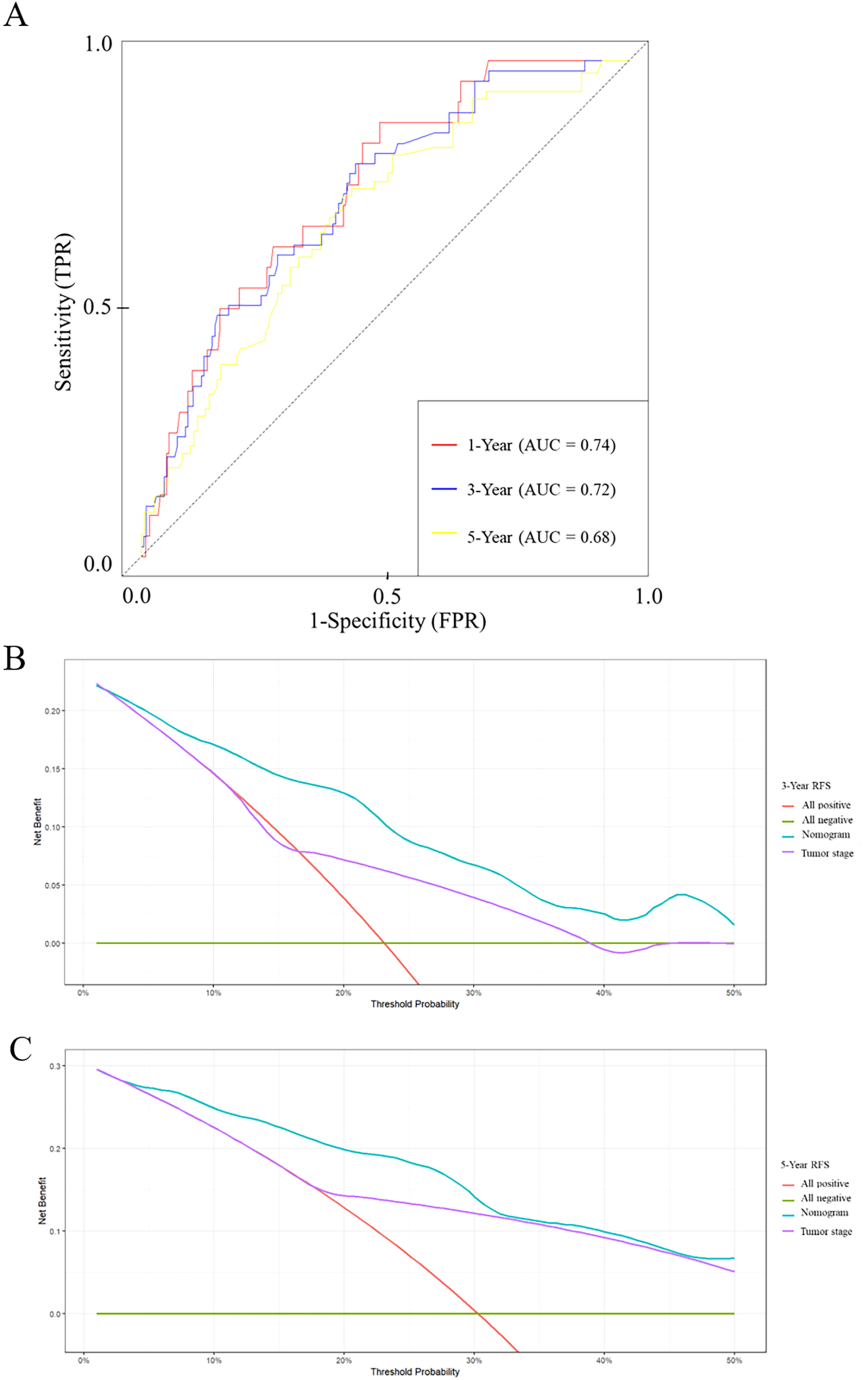
ROC curves and decision curve analyses of the nomogram for RFS prediction. ROC curves for RFS **(A)**. **(B, C)** Decision curve analyses for 3-year **(C)** and 5-year **(D)** RFS prediction. ROC, receiver operating characteristic; RFS, recurrence-free survival; GLR, gamma-glutamyl transpeptidase to lymphocyte count ratio.

Time-dependent ROC curve analysis revealed that the nomogram had areas under the curve (AUCs) for predicting 1-, 3-, and 5-year RFS of 0.74, 0.72, and 0.68, respectively (**Figure 5A**), confirming its stable predictive efficacy at different time points.

Furthermore, decision curve analysis (DCA) demonstrated that, across a wide range of threshold probabilities (5% to 50%), using our nomogram for decision-making provided a greater net clinical benefit than either the ‘treat-all’ or ‘treat-none’ strategies, and was also superior to using tumor stage alone for decision-making (**Figures 5B, C**).

### 3.4 Subgroup analyses

**Figure 6:**
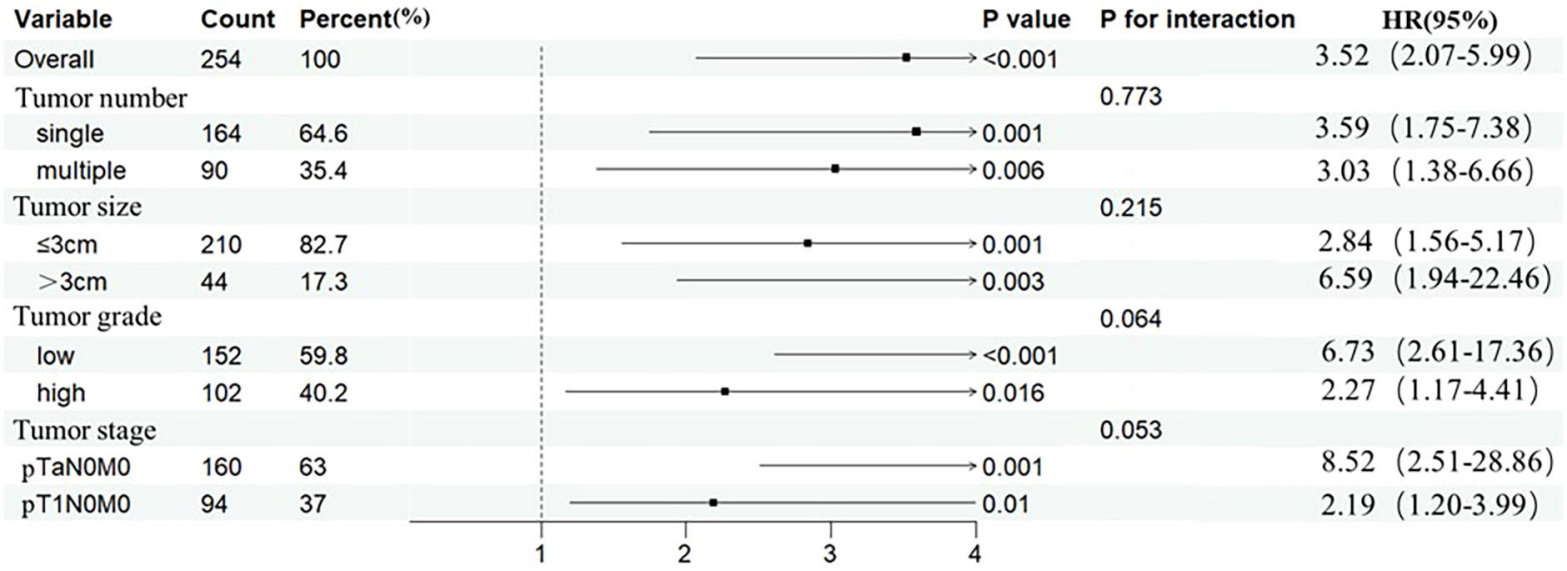
Forest plots for GLR with RFS. RFS, recurrence-free survival; GLR, gamma-glutamyl transpeptidase to lymphocyte count ratio.

To assess the robustness of GLR’s prognostic value, we conducted subgroup analyses. The forest plot (**Figure 6**) visually demonstrates that a high GLR was consistently associated with a significantly increased risk of recurrence across all subgroups stratified by tumor size (≤3 cm vs. > 3 cm), number (single vs. multiple), grade (low vs. high), and stage (Ta vs. T1) (all HR > 1, P < 0.05).

The results of the interaction tests indicated no significant interactions between the prognostic effect of GLR and tumor size (P for interaction = 0.215), number (P for interaction = 0.773), grade (P for interaction = 0.064), or stage (P for interaction = 0.053). This suggests that the prognostic value of GLR as an independent factor is broadly applicable across these different clinicopathological settings.

## 4 Discussion

Non-muscle-invasive bladder cancer (NMIBC) is clinically characterized by its high recurrence rate, posing a significant challenge to long-term patient management. Therefore, the development of simple, reliable, and cost-effective prognostic biomarkers to achieve precise risk stratification is a current research focus in the field of urologic oncology. While other inflammation-based prognostic markers, such as the neutrophil-to-lymphocyte ratio (NLR) and platelet-to-lymphocyte ratio (PLR), have demonstrated prognostic utility in bladder cancer (20), our study highlights the superior predictive value of GLR. Specifically, in our ROC analysis, GLR achieved an AUC of 0.716, significantly outperforming both NLR (AUC = 0.552) and PLR (AUC = 0.535). This performance advantage suggests that GLR is not merely another inflammatory marker but a unique composite index integrating a key metabolic stress indicator (GGT). This composite nature may allow it to capture a more comprehensive picture of the host-tumor interaction, potentially offering distinct prognostic information beyond pure inflammatory cell counts. This study, for the first time, systematically reveals the independent predictive value of the preoperative gamma-glutamyl transferase to lymphocyte ratio (GLR) for postoperative recurrence in NMIBC patients following TURBt. Our core finding is that a high preoperative GLR level is closely associated with a significantly shorter recurrence-free survival (RFS). More importantly, we integrated this novel biomarker with several other independent risk factors to construct and validate a nomogram model with superior predictive performance and clinical utility.

The robust prognostic power of GLR as a composite biomarker is rooted in its simultaneous reflection of two core pathological processes in the tumor microenvironment: metabolic reprogramming and host immunosuppression. From a metabolic perspective, elevated GGT levels serve not only as a marker of systemic oxidative stress but also as a key mechanism for tumor cell adaptation. First, GGT functions as a critical cytoprotective enzyme. By catalyzing the hydrolysis of extracellular glutathione (GSH) and facilitating cysteine recovery, GGT maintains high intracellular GSH pools (21). This mechanism neutralizes oxidative stress induced by surgical trauma and, crucially, facilitates the detoxification of electrophilic chemotherapeutics, such as pirarubicin (22). Specifically, GSH conjugates with these drugs to form thiol-drug conjugates, which are subsequently effluxed by multidrug resistance proteins (MRPs), thereby conferring multidrug resistance (MDR) to residual micrometastases and diminishing the efficacy of intravesical therapy (23). Second, GGT exerts pro-oxidant, tumorigenic effects. The GGT-mediated catabolism of cysteinyl-glycine triggers the Fenton reaction in the presence of transition metals, generating extracellular reactive oxygen species (ROS). This persistent, low-level oxidative stress induces DNA damage and genomic instability in the adjacent, histologically normal urothelium—a process known as “field cancerization”—creating a microenvironment conducive to the evolution of new tumor clones (24).

This biological rationale is strongly supported by clinical evidence across various malignancies. For instance, Yang et al. (25) reported that in resectable esophageal squamous cell carcinoma, patients in the high-GGT risk group had a 1.568-fold higher risk of death and a 1.582-fold higher risk of recurrence compared to the low-risk group. In the field of uro-oncology, Georgios Gakis et al. (26) similarly found that elevated preoperative serum GGT was independently associated with increased all-cause mortality following radical cystectomy. These findings align with our group’s previous research, which demonstrated that elevated preoperative GGT is an independent risk factor for poorer Overall Survival (OS), Disease-Free Survival (DFS), and Cancer-Specific Survival (CSS) in bladder cancer patients undergoing radical cystectomy, a conclusion further validated by our subsequent meta-analysis (13, 27). Crucially, the current study extends this principle from advanced, muscle-invasive disease to the earlier, non-muscle-invasive stage of bladder cancer.

On the other hand, the peripheral blood lymphocyte count (the denominator of GLR) serves as a surrogate marker for systemic immune competence (28). Lymphopenia typically signifies compromised immune surveillance, allowing residual micrometastases or newly formed tumor cells to evade immune clearance (14). When the GGT-mediated “metabolic and resistance advantage” is coupled with the “immune deficit” indicated by lymphopenia, a pathological state highly prone to recurrence is established.

Our multivariate Cox regression analysis provides robust confirmation of this theory. Even after adjusting for potent traditional predictors such as tumor size, number, grade, and stage, the risk of recurrence in the high-GLR group remained nearly three times that of the low-GLR group (HR = 2.822). This suggests that the “metabolic-immune mismatch” reflected by GLR is a core prognostic factor, independent of local tumor characteristics. This finding resonates with the growing body of evidence highlighting the critical prognostic role of systemic inflammation and metabolism in advanced bladder cancer (29), suggesting that these fundamental biological processes are relevant across all stages of the disease.

A noteworthy finding of this study is that GLR was significantly associated with NMIBC recurrence but not with overall survival (OS) (P = 0.33). This finding likely reflects the distinct natural history of NMIBC. Post-TURBt recurrence is predominantly a local, non-lethal event, whereas OS in this older cohort (median age 67) is substantially influenced by competing risks of mortality from age-related comorbidities. Therefore, GLR appears to be a more specific biomarker for the biological drivers of tumor recurrence rather than a predictor of all-cause mortality. This suggests that GLR may more specifically reflect the biological mechanisms driving local tumor recurrence rather than those leading to systemic disease progression and death.

To translate the prognostic value of GLR into a clinically practical tool, we constructed a multi-factor prognostic nomogram. The superior performance of this model is demonstrated in several aspects. First, in terms of predictive accuracy, the model exhibited excellent discrimination with a concordance index (C-index) of 0.785. Critically, compared to a baseline model without GLR (C-index = 0.745), the addition of GLR increased the C-index by 0.04, an incremental value that demonstrates GLR provides unique prognostic information beyond traditional pathological factors. Second, regarding reliability, the calibration curves showed a high degree of consistency between the nomogram-predicted probabilities and the actual observed frequencies at 3 and 5 years, and the model’s robustness was confirmed by internal validation with 1000 bootstrap resamples. Finally, regarding clinical utility, decision curve analysis (DCA) showed that across a wide range of clinical decision thresholds (5% to 50%), the net benefit of using our nomogram for decision-making was significantly higher than the ‘treat-all’/’treat-none’ strategies or relying on a single indicator like tumor stage. This indicates that the nomogram can serve as a valuable tool to refine individualized risk stratification, thereby aiding clinicians and patients in making more informed decisions regarding the intensity and frequency of follow-up surveillance or the consideration of adjuvant therapies. This means it can effectively help clinicians avoid unnecessary interventions and identify true high-risk patients.

A key strength of this study is the rigorous assessment of potential confounding variables that commonly affect biomarker research. We specifically investigated the influence of postoperative adjuvant therapy and alcohol consumption history. Our analysis demonstrated that the allocation of adjuvant therapies (BCG vs. intravesical chemotherapy) was balanced between the high and low GLR groups (P = 0.844), which mitigates the risk of treatment selection bias. Furthermore, in univariate analysis, neither the type of adjuvant therapy (P = 0.429) nor a history of alcohol consumption (P = 0.110) emerged as a significant predictor of recurrence. These crucial findings provide strong evidence that the prognostic power of GLR is not merely a reflection of these external factors. Instead, they support the hypothesis that GLR captures an intrinsic biological state of host-tumor interaction, thereby enhancing confidence in its utility as a robust and independent biomarker. Furthermore, our model identified a ‘history of prior abdominal surgery’ as a novel independent risk factor. While the underlying mechanisms require elucidation, several hypotheses warrant investigation, including surgery-induced chronic low-grade inflammation, alterations in microbial immunomodulation via the ‘gut-bladder axis’, or its function as a surrogate marker for overall patient frailty (30, 31). Furthermore, our model innovatively identified a ‘history of prior abdominal surgery’ as an independent risk factor for recurrence. Although the precise mechanism remains to be elucidated, we speculate this is not a coincidental finding and may be related to surgery-induced chronic low-grade inflammation, alterations in microbial immunomodulation via the ‘gut-bladder axis’, or its role as a surrogate marker for poorer overall patient health (30, 31).

Despite these strengths, this study has some limitations. First and foremost, its retrospective, single-center design inherently carries risks of selection bias and limits the generalizability of our findings. Therefore, large-scale, multicenter prospective validation is essential before these results can be widely applied. Second, the utilization rate of Bacillus Calmette-Guérin (BCG) immunotherapy in our cohort was relatively low (4.3%). This is primarily attributed to the specific policies of the national medical insurance system in China, where BCG is classified as a non-reimbursable treatment. To alleviate the significant out-of-pocket financial burden, the vast majority of patients with intermediate- to high-risk NMIBC or CIS opted for pirarubicin-based chemotherapy, which is fully covered by insurance. Although we adjusted for the type of adjuvant therapy in our multivariate analysis, the small sample size of the BCG group limits the statistical power to fully evaluate the specific interaction between GLR and BCG response. Future large-scale studies are warranted to validate the prognostic value of GLR across different adjuvant treatment modalities. Third, while we successfully controlled for alcohol consumption—a major potential confounder for GGT—we were unable to collect detailed data on other factors such as specific medication history or the presence of non-malignant liver conditions. These unmeasured factors may have introduced some residual bias into the GLR values. Finally, the GLR was measured at a single preoperative time point. This static measurement may not capture the dynamic nature of the host’s inflammatory and metabolic state. Future prospective studies should aim to explore the prognostic value of serial GLR monitoring.

In conclusion, this study provides strong initial evidence that preoperative GLR is a novel, simple, and powerful independent biomarker for predicting postoperative recurrence in NMIBC patients. The GLR-based nomogram offers a promising tool for individualized risk stratification. However, looking forward, several critical steps are necessary before this biomarker can be integrated into routine clinical practice. Future research should prioritize the prospective validation of our findings in large, multicenter, and diverse cohorts. Such studies are essential to confirm the robustness and generalizability of the GLR cutoff value and the nomogram’s performance. Furthermore, future prospective studies should be designed to include the serial measurement of GLR at multiple time points, such as post-treatment and during follow-up surveillance. Investigating the trajectory of GLR (e.g., whether a rising GLR post-treatment predicts imminent recurrence) could provide a dynamic monitoring tool that offers significantly more clinical utility than a single preoperative measurement. The development of user-friendly tools, such as an online calculator, will also be crucial for facilitating clinical integration once external validation is complete.

## 5 Conclusion

Preoperative serum GLR is an independent prognostic biomarker for RFS in patients undergoing transurethral resection of bladder tumor for NMIBC. Our GLR-based nomogram provides a more precise model for individualized risk stratification, holding significant potential to guide personalized surveillance and optimize adjuvant treatment decisions.

## Data Availability

The raw data supporting the conclusions of this article will be made available by the authors, without undue reservation.

## Supplementary material

Supplementary Table 1 Baseline characteristics of patients stratified by overall survival (OS) status.

Supplementary Table 2 Univariate and multivariate Cox regression analysis for overall survival (OS).
