# Supplementary material for: Preoperative gamma-glutamyl transferase to lymphocyte ratio predicts recurrence in non-muscle-invasive bladder cancer
Source: Front Oncol. 2026 Feb 10;16:1724968. doi: 10.3389/fonc.2026.1724968 (PMC12929151; doi:10.3389/fonc.2026.1724968)
Supplement: Supplementary Table 1 — Baseline characteristics of patients stratified by overall survival (OS) status. [file Table1.docx]

Supplementary Material

# Supplementary Figures and Tables

## Supplementary Tables

Supplementary Table 1 Baseline characteristics of patients stratified by overall survival (OS) status

| Characteristics | Alive | Deceased | P-value |
| --- | --- | --- | --- |
|  | (n=232) | (n=22) |  |
| Age (years) (mean ± SD) | 64.9 ± 12.3 | 76.8 ± 5.9 | <0.001 |
| GLR (mean ± SD) | 13.8 ± 8.5 | 15.3 ± 10.0 | 0.821 |
| NLR (mean ± SD) | 2.6 ± 1.7 | 3.0 ± 2.2 | 0.764 |
| PLR (mean ± SD) | 130.4 ± 49.9 | 122.43 ± 38.3 | 0.457 |
| Gender, n (%) |  |  | 0.213 |
| Female | 31 (13.4%) | 5 (22.7%) |  |
| Male | 201 (86.6%) | 17 (77.3%) |  |
| History of abdominal surgery, n (%) |  |  | 0.749 |
| No | 201 (86.6%) | 20 (90.9%) |  |
| Yes | 31 (13.4%) | 2 (9.1%) |  |
| Hypertension, n (%) |  |  | 0.434 |
| No | 136 (58.6%) | 11 (50.0%) |  |
| Yes | 96 (41.4%) | 11 (50.0%) |  |
| Diabetes, n (%) |  |  | 0.096 |
| No | 203 (87.5%) | 16 (72.7%) |  |
| Yes | 29 (12.5%) | 6 (27.3%) |  |
| Coronary heart disease, n (%) |  |  | 0.293 |
| No | 207 (89.2%) | 18 (81.8%) |  |
| Yes | 25 (10.8%) | 4 (18.2%) |  |
| Smoking, n (%) |  |  | 0.208 |
| No | 160 (69.0%) | 18 (81.8%) |  |
| Yes | 72 (31.0%) | 4 (18.2%) |  |
| Drinking, n (%) |  |  | 0.172 |
| No | 98(42.2%) | 6(27.3%) |  |
| Yes | 134(57.8%) | 16(72.7%) |  |
| Tumor number, n (%) |  |  | 0.135 |
| Single | 153 (65.9%) | 11 (50.0%) |  |
| Multiple | 79 (34.1%) | 11 (50.0%) |  |
| Tumor size, n (%) |  |  | 0.554 |
| ≤3cm | 193 (83.2%) | 17 (77.3%) |  |
| ＞3cm | 39 (16.8%) | 5 (22.7%) |  |
| Tumor grade, n (%) |  |  | <0.001 |
| Low | 147 (63.4%) | 5 (22.7%) |  |
| High | 85 (36.6%) | 17 (77.3%) |  |
| Tumor stage, n (%) |  |  | <0.001 |
| Ta | 154 (66.4%) | 6 (27.3%) |  |
| T1 | 78 (33.6%) | 16 (72.7%) |  |
| Concomitant CIS |  |  | 0.005 |
| No | 230 (99%) | 19 (86%) |  |
| Yes | 2 (0.9%) | 3 (14%) |  |
| Postoperative adjuvant therapy, n (%) |  |  | 0.899 |
| Intravesical chemotherapy | 220(94.8%) | 21(95.5%) |  |
| BCG | 12(5.2%) | 1(4.5%) |  |
