# Supplementary material for: Preoperative gamma-glutamyl transferase to lymphocyte ratio predicts recurrence in non-muscle-invasive bladder cancer
Source: Front Oncol. 2026 Feb 10;16:1724968. doi: 10.3389/fonc.2026.1724968 (PMC12929151; doi:10.3389/fonc.2026.1724968)
Supplement: Supplementary Table 2 — Univariate and multivariate Cox regression analysis for overall survival (OS). [file Table2.docx]

Supplementary Material

# Supplementary Figures and Tables

## Supplementary Tables

Supplementary Table 2 Univariate and multivariate Cox regression analysis for overall survival (OS)

| Characteristic | Univariate Analysis | | Multivariate Analysis | |
| --- | --- | --- | --- | --- |
|  | Hazard Ratio(95%CI) | P value | Hazard Ratio(95%CI) | P value |
| Gender |  |  |  |  |
| Female | Reference |  |  |  |
| Male | 0.501(0.183-1.369) | 0.178 |  |  |
| Age | 1.114(1.059-1.172) | ＜0.001 | 1.105(1.038-1.176) | 0.002 |
| Diabetes |  |  |  |  |
| No | Reference |  |  |  |
| Yes | 2.061(0.753-5.644) | 0.159 |  |  |
| History of abdominal surgery |  |  |  |  |
| No | Reference |  |  |  |
| Yes | 0.861(0.200-3.708) | 0.841 |  |  |
| Hypertension |  |  |  |  |
| No | Reference |  |  |  |
| Yes | 1.344(0.582-3.103) | 0.489 |  |  |
| Coronary heart disease |  |  |  |  |
| No | Reference |  |  |  |
| Yes | 1.256(0.412-3.823) | 0.688 |  |  |
| Smoking |  |  |  |  |
| No | Reference |  |  |  |
| Yes | 0.551(0.185-1.638) | 0.284 |  |  |
| Drinking |  |  |  |  |
| No | Reference |  |  |  |
| Yes | 1.472(0.570-3.801) | 0.424 |  |  |
| Tumor number |  |  |  |  |
| Single | Reference |  | Reference |  |
| Multiple | 2.158(0.916-5.086) | 0.079 | 1.626(0.669-3.952) | 0.283 |
| Tumor size |  |  |  |  |
| ≤3cm | Reference |  |  |  |
| ＞3cm | 1.723(0.628-4.728) | 0.291 |  |  |
| Tumor grade |  |  |  |  |
| Low | Reference |  | Reference |  |
| High | 6.335(2.131-18.835) | 0.001 | 3.401(1.109-10.430) | 0.032 |
| Tumor stage |  |  |  |  |
| Ta | Reference |  | Reference |  |
| T1 | 3.571(1.379-9.248) | 0.009 | 2.436(0.904-6.561) | 0.078 |
| Concomitant CIS |  |  |  |  |
| No | Reference |  | Reference |  |
| Yes | 12.575(3.656-43.351) | ＜0.001 | 3.150(0.819-12.120) | 0.095 |
| GLR group |  |  |  |  |
| ≤11.71 | Reference |  |  |  |
| ＞11.71 | 0.652(0.273-1.555) | 0.335 |  |  |
| Postoperative adjuvant therapy, n (%) |  |  |  |  |
| Intravesical chemotherapy | Reference |  |  |  |
| BCG | 1.337(0.176-10.159) | 0.779 |  |  |
